# Recurrent transient global amnesia related to dural arteriovenous fistula

**DOI:** 10.1590/1980-5764-DN-2024-0222

**Published:** 2025-06-02

**Authors:** Rafael Batista João, Raquel Filgueiras Mattos

**Affiliations:** 1 Hospital Municipal Dr. Jose de Carvalho Florence, Departamento de Neurologia, São José dos Campos SP, Brazil.; 2 Instituto Neurológico de Goiânia, Departamento de Neurologia, Goiânia GO, Brazil.

**Keywords:** Amnesia, Transient Global, Amnesia, Arteriovenous Fistula, Hyperemia, Vascular Malformations, Amnésia Global Transitória, Amnésia, Fístula Arteriovenosa, Hiperemia, Malformações Vasculares

## Abstract

Transient global amnesia (TGA) is a syndrome characterized by the sudden onset of anterograde amnesia, typically lasting for a few to less than 24 h. This condition is considered benign, as it is usually self-limited and unrelated to brain lesions; however, TGA may less frequently occur due to vascular brain changes. We report a rare case of a 66-year-old male with a diagnosis of recurrent TGA associated with a brain dural arteriovenous fistula (DAVF), alongside the absence of clear triggering and risk factors. He was submitted to embolization and remained event-free until his last attempt, 18 months after the surgical procedure. The case presentation follows a brief literature review on TGA and the pathophysiological mechanisms probably underpinning its occasional correlation with brain DAVF.

## RESUMO.

A amnésia global transitória (AGT) é uma síndrome caracterizada pelo início súbito de amnésia anterógrada, que tipicamente dura de algumas horas a até menos de 24 horas. Essa condição é considerada benigna, pois geralmente é autolimitada e não está relacionada a lesões cerebrais, no entanto a AGT pode, com menor frequência, ocorrer por causa de alterações vasculares cerebrais. Apresentamos o raro caso de um homem de 66 anos de idade com diagnóstico de AGT recorrente associada a uma fístula arteriovenosa dural cerebral (FAVD), concomitantemente à ausência de evidentes fatores desencadeantes ou de risco. O paciente foi submetido à embolização e permaneceu livre de novos eventos até seu último seguimento, 18 meses após o procedimento cirúrgico. A apresentação do caso é seguida de uma breve revisão de literatura sobre AGT e os mecanismos fisiopatogênicos que provavelmente explicam sua ocasional correlação com a FAVD cerebral.

## INTRODUCTION

A cute episodes of cognitive changes are challenging in clinical practice, and transient global amnesia (TGA) is one of the main possible diagnoses in this setting^1^. The main clinical presentation of TGA is a sudden onset of anterograde amnesia of short duration (typically less than 24 h) with spared self-identification. TGA is usually benign and self-limited^1,2^; however, some cases may occur secondarily to brain vascular abnormalities^3,4^. Thus, we report and discuss a case of brain dural arteriovenous fistula (DAVF) diagnosed after recurrent episodes of TGA in a male.

For case discussion and literature review, we selected relevant publications from widely known databases (PubMed Central^®^, SciELO, Google Scholar, Europe PubMed Central^®^, and Wiley Online Library) using the following terms: Transient Global Amnesia, Global Transient Amnesia, Amnesia, and Dural Arteriovenous fistula.

## CASE REPORT

A Caucasian 66-year-old right-handed male with a previous history of hypertriglyceridemia (in use only of ezetimibe) was evaluated in an outpatient clinic after two previous episodes of transient amnesia, separated by 4 weeks. The events were witnessed by his wife, who described manifestations compatible with the sudden onset of amnesia with preserved self-identity and repetitive similar questions. In both episodes, these clinical manifestations occurred for approximately 4 h and were followed by spontaneous total recovery (last event: 3 weeks before the first consultation). She reported no observation of consciousness impairment, starring, automatisms, tonic or dystonic postures, clonic movements, head and ocular version, drooling, skin color changes, sphincter dysfunction, or any signs of paresis. The patient denied medication use, drug/substance abuse or any symptoms preceding the episode, a personal or familial history of migraine, epileptic seizures, or psychiatric disorders. He added that the second event occurred just after sexual intercourse (not associated with strenuous physical exercise). His general and neurological examinations were normal at the consultations. Additionally, he underwent cognitive assessments, including the Montreal Cognitive Assessment, scoring 30/30, and delayed recall tests (e.g., word list recall and logical memory test), which were unremarkable.

**Figure 1:**
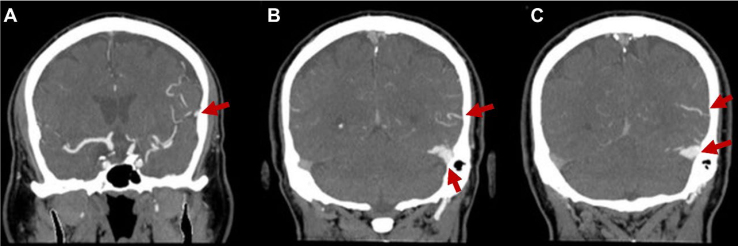
Brain AngioCT coronal sections showing: (A) dilated vessels arising from the distal segment of the left posterior cerebral artery (arrow); (B) abnormal arteriovenous communication involving the left posterior cerebral artery circulation and the posterior ipsilateral transverse sinus (arrows); (C) arterialized venous drainage into the transverse sinus during the arterial phase, confirming the presence of the dural arteriovenous fistula (arrows).

**Figure 2:**
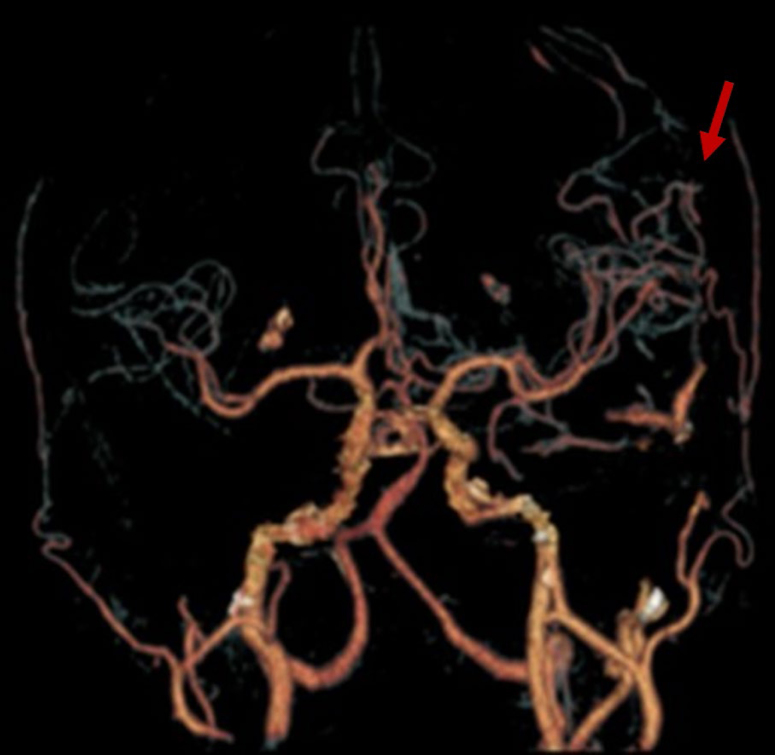
Brain AngioCT tridimensional reconstruction showing a left dural arteriovenous fistula involving mainly the posterior arterial and venous circulations (arrow).

Considering his age and risk factors, we started a complementary investigation focusing mainly on vascular etiologies. The electroencephalograms (one register with 20-min and one with 40-min duration) and the transthoracic echocardiogram were unremarkable, and the Holter showed sporadic ventricular extrasystoles. Because claustrophobia limited the acquisition of magnetic resonance imaging (MRI), he was initially assessed with brain computed tomography (CT) and computed angiotomography (AngioCT) of the brain and cervical vessels, aiming additional assessments of vascular structural etiologies (e.g., carotid stenosis, arteriovenous malformations, and DAVF). The brain CT was unremarkable, and the AngioCT showed a DAVF located on the distal segment of the left posterior cerebral artery communicated to the distal third of the left transverse sinus (with early enhancement during the arterial phase); there was also early enhancement of the inferior Labbé vein, superior Trolard vein, and superficial cortical veins on the fronto-temporo-parietal region (the main radiological findings are shown in Figures 1 and 2). He was then sent to neurovascular intervention evaluation and posteriorly submitted to embolization. During the follow-up, he denied new similar events until his last consultation, 18 months after the procedure.

## DISCUSSION

In 1964, TGA gained notoriety with the work of Fisher and Adams^5^, despite having been previously described by Bender^6^ and independently by Guyotat and Courjon^7^ in 1956. Only in 1990, a diagnostic criterion for TGA was initially proposed by Caplan^8^ and later consolidated by Hodges and Warlow^9^. Although clinical criteria were proposed and even after a long time dedicated to the TGA investigation, the exact pathophysiological mechanisms are still unexplained^10^.

We consider that our patient presented typical features of TGA, as the current diagnostic criteria for this condition include the following^9^:

- The attack must be witnessed and information available from a capable observer who was present for most of the attacks;
- Clear-cut anterograde amnesia during the attack;
- Cognitive impairment limited to amnesia, without clouding of consciousness or loss of personal identity;
- No accompanying focal neurologic symptoms during the attack and no significant neurologic signs afterward;
- Absence of epileptic features;
- Resolution of the attack within 24 h;
- Patients with recent head injury or active epilepsy are excluded.

TGA commonly occurs in the seventh life's decade (with a mean age ranging from 61 to 67.3 years)^1^, and is often associated with triggering factors, such as physical exercise, intense emotions, sexual intercourse, Valsalva maneuver^11^, environmental temperature changes^12^, and acute pain^13^. A personal and/or family history of migraine is recognized as a risk factor^14,15^. A study including a pooled analysis of 1333 TGA cases showed no statistically significant gender prevalence (46.4% men and 53.6% women; p=0.49)^16^.

Usually, TGA is described as a benign and unique event^17^; however, some cases may be recurrent^18,19^. A recently published meta-analysis including 36 observational case–control and cohort studies assessed 4,514 TGA cases and showed a proportion of 12.73% of recurrent events. The same study identified the following statistically significant recurrent TGA factors: depression (odds ratio [OR]=4.4871; 95% confidence interval [CI] 1.890–10.651; p=0.0288), personal history or current state of migraine (OR=2.0795; 95%CI 1.3892–3.1128; p=0.003), and sexual intercourse before the event (OR=1.481; 95%CI 1.0341–2.1222; p=0.04)^19^. Beyond clinical factors, recurrent TGA may be associated with identifiable structural abnormalities, such as DAVF, in which venous congestion may partially underpin the physiopathogenic mechanism^4^.

Other than venous congestion, classic theories have been put forward to explain the mechanism of TGA^16^. Cortical spreading depression (CSD), a wave of transient neuronal and glial depolarization followed by the suppression of neuronal activity, is supposed to be involved. This process can lead to a transient inhibition of hippocampal function especially in the CA1 field of the hippocampus, which is very sensitive to metabolic insults^20^. In addition, brain MRI studies have shown hippocampal and other limbic system structures’ diffusion restriction during TGA attacks, indicating that transient ischemia or perfusion changes may be associated with this condition^21–23^. Neuronal metabolism impairment affecting hippocampal function has also been demonstrated. In this line, a study using magnetic resonance spectroscopy to investigate metabolic changes in the hippocampal CA1 area during an acute TGA attack showed increased lactate levels as a marker of anaerobic glycolysis during metabolic stress^24^. Glutamatergic excitotoxicity, occurring during metabolic stress or ischemia, may also lead to temporary neuronal dysfunction in this context^25^. In TGA cases correlated with DAVF, dynamic blood flow changes, and these mechanisms likely interact^3,4,22–25^.

Two reports in the literature described the coexistence of TGA and DAVF^3,4^. As in our current case, a similarly good outcome was reported after vascular intervention in the first registered case by Takasahi et al. This study cited important vascular mechanisms leading to the occurrence of TGA, including carotid dysfunction and DAVF (as a source of venous congestion), affecting the frontal and temporal lobes^3^. In this setting, one classic paper suggested that some cases of TGA could be a consequence of the transient retrograde transmission of venous blood flow from the superior vena cava tributaries through incompetent valves to the cerebral venous system,^26^ leading mainly to impairment of the thalamus, diencephalon, and mesial temporal structures (as the hippocampus)^26,27^. This observation may partially explain the correlation between physical exercise and Valsalva-like maneuvers as TGA triggers in some cases^28–30^, as increases in intrathoracic pressure may result in jugular hypertension and thus in abnormal venous flux to the brain veins and sinuses^31,32^. Hypothetically, this process could cause either CSD or ischemia in specific brain structures^33^. In fact, our patient presented no more new episodes of transient amnesia 18 months after the DAVF embolization, probably because of venous flow normalization.

The transitory dysfunction of the hippocampus (especially in the CA1 field of the cornu ammonis) plays a pivotal role in the pathogenesis of TGA^34^. This structure is drained by several veins, including the hippocampal veins^35^. The venous drainage pattern can vary among individuals, but, typically, the hippocampal veins drain into the cerebral veins, such as the basal vein of Rosenthal and the great vein of Galen^36^. Ultimately, the blood from these venous structures is collected by the internal cerebral veins, which then drain into the dural venous sinuses, such as the straight sinus and transverse sinuses^37^. In the current case, the brain AngioCT showed communication of the posterior cerebral artery to the left transverse sinus. In 2018, Johnson et al.^4^ reported the case of a female with a history of two episodes of mental confusion and anterograde amnesia (the last one triggered during physical exercise). Her brain magnetic resonance angiography showed a DAVF supplied by branches of the vertebral and posterior cerebral arteries draining into the vein of Galen.

Furthermore, other reports also associated DAVF with cognitive impairment, but not precisely TGA^38–40^. In 2008, Gonçalves et al. published the case of a 43-year-old male who presented a subacute onset of apathy and memory deficits with progressive worsening secondary to a bilateral thalamic lesion associated with an anterior cranial fossa DAVF^38^. In 2009, Lantz et al. reported a case of acute confusion triggered by the Valsalva maneuver in a 67-year-old male, who had a right parietal DAVF identified after MRI and angiography^39^. In 2013, Iwasawa et al. described the case of a 54-year-old male with memory deficits and apathy decline for 3 months secondary to bilateral thalamic lesions caused by a DAVF at right transverse sigmoid sinuses, leading to flow impairment in the vein of Galen^40^. Interestingly, all these patients presented significant improvement in cognitive status after the embolization^38–40^. Similarly, we also believe that the cognitive changes reported during the episodes in the case of our patient could be partially related to venous congestion transiently affecting thalamic function. Thus, although routine assessments of vascular etiology in all suspected structural transient amnesia cases may not be mandatory, complement with angiographic studies beyond MRI evaluation could add accuracy to the investigation, mainly when episodes reoccur associated with possible dynamic hemodynamic changes.

Some limitations are notable in our report. First, the lack of a brain MRI possibly showing a hippocampal diffusion restriction ipsilaterally to the DAVF precluded a more solid characterization of the case as TGA; however, the first consultation occurred 3 weeks after the last transient amnesia episode. In this period, such brain MRI changes could not be present anymore. Second, the temporal lobe impairment due to the vascular changes identified could increase the chance of transient epileptic amnesia as a possibility; nonetheless, there were no typical clinical or electrophysiologic epileptic manifestations during the follow-up. Third, although there is a possibility that TGA occurred by chance alongside the presence of a DAVF, there were no more new episodes 18 months after the surgical procedure, which led us to consider the probable association between these conditions.

Finally, research has shown the importance of DAVF as a cause of TGA and other cognitive syndromes. Due to its rarity and the need for rapid intervention, the literature is still lacking about which specific cognitive deficits may occur due to this condition^32^. In addition, we hypothesize that some cases of TGA related to venous congestion could be underreported in the literature. An increase in the number of studies concerning this topic could help health professionals be aware of the possibility of vascular abnormalities as potentially treatable causes of cognitive entities.

In conclusion, despite the classical description of TGA as benign, the complementary investigation may show risky but potentially treatable causes under reasonable suspicion. In addition, venous congestion may be the main physiopathogenic mechanism in some cases, especially in those related to transitory pressure elevations in abdominal and thoracic cavities. The association between TGA and DAVF is very rare, and further studies are necessary on this topic.
