# Erratum: Recurrent transient global amnesia related to dural arteriovenous fistula

**DOI:** 10.1590/1980-5764-DN-2024-0222-ER

**Published:** 2025-12-01

In the article "Recurrent transient global amnesia related to dural arteriovenous fistula", DOI https://doi.org/10.1590/1980-5764-DN-2024-0222, published in the Dement Neuropsychol 2025;19:e20240222

**Where it was written:**

Raquel Filgueiras Mattos

**It should be:**

Raquel Mattos Filgueiras

Editor-in-Chief: Sonia M. D. Brucki http://orcid.org/0000-0002-8303-6732

